# Investigating spatiotemporal dynamics and trade-off/synergy of multiple ecosystem services in response to land cover change: a case study of Nanjing city, China

**DOI:** 10.1007/s10661-020-08663-x

**Published:** 2020-10-13

**Authors:** Jie Zhao, Cheng Li

**Affiliations:** 1 Belt & Road Institute, Jiangsu Normal University, Xuzhou, 221009 China; 2 School of Architecture & Design, China University of Mining and Technology, DaXue Road 1, Xuzhou, 221116 Jiangsu China

**Keywords:** Ecosystem services, Trade-offs, Land cover change, Markov-CA model, Scenarios, Urban planning

## Abstract

Investigating the spatiotemporal trends and trade-off/synergy relationships among ecosystem services can provide effective support for urban planning and decision making toward sustainable development. With Nanjing city in China as a case study, this study assessed the spatiotemporal dynamics of six key ecosystem services from 2005 to 2030. Integration of Markov-cellular automata and ecosystem services models was realized to analyze the potential impacts of future urbanization on ecosystem services by simulating business-as-usual (BAU), cropland protection (CP), and ecological restoration (ER) scenarios. Furthermore, an innovative trade-off/synergy degree was developed to quantify the magnitude of the complex relationship among the multiple ecosystem services under the different scenarios. Due to the rapid expansion of built-up land, carbon storage, habitat quality, and air purification decreased 2.92%, 5.80%, and 7.91%, respectively. The CP scenario exhibited the highest crop production values, and the ER scenario was a better urban development strategy that enhanced the regulating ecosystem services at the expense of crop production. To promote urban ecosystem services and minimize trade-offs, we proposed certain future urban development strategies, including ecological corridor construction and compact development. The study could provide a scientific reference for the effective ecosystem management of Nanjing and other rapidly urbanized regions.

**Electronic supplementary material:**

The online version of this article (10.1007/s10661-020-08663-x) contains supplementary material, which is available to authorized users.

## Introduction

Ecosystem services refer to the direct or indirect products and services obtained from ecosystems that are prerequisites for the survival, health, and livelihood of the human population (Raudsepp-Hearne et al. 2010). Ecosystem services can be divided into four types: supporting, provisioning, regulating, and cultural services, which are crucial for human well being (Costanza et al. 1997; Kragt and Robertson 2014). According to the Millennium Ecosystem Assessment (MEA) report, approximately 60% of the world’s ecosystem services exhibit a degradation trend, which have a serious impact on human well-being and directly threaten the sustainable development (MEA 2005). This is especially the case in urban area, where the ecosystem services experienced significant degradation due to the intensifying ecosystem-humanity interactions (Schröter et al. 2005). In recent decades, the world has experienced rapid urbanization, with an urban population accounting for 29.5% of the total population in 1950, increasing to 55.3% in 2018 (United Nations 2019). This rapid growth trend is likely to continue without any signs of slowing down. The urbanization level in the world is predicted to reach 60.5% by 2030 (United Nations 2019) and that 60% of the land that will convert to built-up land in 2030 has yet to be developed (Elmqvist et al. 2013). Although urbanization notably promotes the improvement of socioeconomic conditions, it simultaneously poses an increasing threat to urban ecological security. It has been widely accepted that urbanization is one of the most important factors that contributed to the degradation in ecosystem services (Costanza et al. 2014; Kindu et al. 2016). During the urbanization process, natural ecological landscapes such as forests, grasslands, and pastoral areas are continuously transformed into human-dominated landscapes at a rapid pace to fulfill the growing requirements of social and economic development. Because the function and structure of ecosystems are altered, the above transformation process has resulted in the loss of urban ecosystem services, such as reduced soil conservation (Sun and Li 2017), decreased carbon storage (He et al. 2016), and degraded air quality (Parsa et al. 2019), and in turn, the deterioration of ecosystem services seriously threatens the survival and health of humans (MEA 2005).

The land cover change induced by urbanization process is exerting pressure to the sustainable provision of urban ecosystem services, and the challenge are expected to be more serious in the context of increasingly intensive human disturbances in urban area. To address this issue, the 11st goal of Sustainable Development Goals (SDGs) proposed by United Nations (UN), Sustainable Cities and Communities, highlighted the necessity to mitigate degradation of urban ecosystem services and coordinate the relationship between human disturbance and urban ecosystem for realizing sustainable urban development (United Nations 2018). How to investigate the ecological effects of land cover change in urban area and mitigate the negative effects on ecosystem services has been a global concern for decision makers.

With an increasing evidence that promoting ecosystem services in urban area can be an effective objective for supporting urban planning and decision making, ecosystem service concept and approach are adopted by governments to address challenges to sustainable urban development (de Groot et al. 2012; Yang et al. 2019). The investigation of spatiotemporal dynamics in ecosystem services and their trade-off/synergy under the impact of urban land cover change can aid in the designing and implementing sustainable urban development policy (Xu et al. 2018). Thus, the study on the spatiotemporal changes of ecosystem services and trade-off/synergy relationships due to urban land cover change induced by urbanization becomes increasingly urgent for facilitating sustainable urban development policy.

There are many studies on the assessment of ecosystem services and the impact of historical land cover change on urban ecosystem services (Anaya-Romero et al. 2016; Wang et al. 2019). By conducting simulations of multiple future land use scenarios, studies have not only assessed the impact of past land cover change on ecosystem services but have also analyzed the change in future ecosystem service values (Lauf et al. 2014; Xie et al. 2018). Scenario analysis has been proven to be effective in assessing the underlying relationship between land cover change and ecosystem services by developing various scenarios related to future development policies. Moreover, the obtained scenario analysis results can provide support for optimal urban planning and ecosystem management in terms of addressing the potential consequences under different development scenarios. Recently, many studies have been conducted to optimize urban planning to protect several key ecosystem services. For example, Gaglio et al. (2019) investigated the effects of biodiversity protection initiatives and the related land cover changes on ecosystem services. Asadolahi et al. (2018) analyzed the trade-off between multiple ecosystem services under land use change scenarios. Delphin et al. (2016) analyzed the impact of future urbanization on the ecosystem services in Florida, USA, and concluded that CS will decrease in the future. Xie et al. (2018) projected the potential impacts of urban expansion on ecosystem services by simulating future scenarios with a case study of Beijing, China. Due to the complexity of urbanization and uncertainty of future urban development, simulating accurate development scenarios and investigating the impacts of future land cover change on urban ecosystem services remain challenging.

In reality, decision makers often wish to maximize one or several types of ecosystem services according to urban livelihood demands, but a principal challenge is that ecosystem services are not independent and may have complex interactions. The situation in which increasing certain ecosystem services may cause a loss in other services has been recognized as a trade-off. For instance, enhancing crop production may cause an increase in the risk of soil erosion (Xu et al. 2016), while Goldstein et al. (2012) found that an increase in carbon storage could lead to a reduction in water quality. The research of Jia et al. (2014) indicated that a trade-off exists between the net primary productivity and water yield. The opposite is true for synergies, which can be defined as situations where both services exhibit a consistent trend. Increasing one or more ecosystem services does not indicate that other ecosystem services will be simultaneously enhanced (Egoh et al. 2008). Understanding the trade-offs and synergies among multiple ecosystem services could help in better understanding the hidden consequences of preferring one ecosystem service over another and achieving sustainable urban development (Hanes et al. 2017; Li and Wang 2018). However, the ecosystem service trade-offs/synergies have largely been ignored. Traditionally, urban planning generally focused on the maximizing some key ecosystem services, such as air quality (Parsa et al. 2019), water purification (Wu et al. 2019), and climate regulation (Gao et al. 2019). These policies and plans overlooked the complex relationship among multiple ecosystem services and fell short of effectively improving overall ecosystem service benefits. Therefore, it is of great significance to examine the trade-off/synergy relationships among ecosystem services and promote the maximization of human well-being to achieve a win-win situation of the regional urban ecosystem and socio-economy. Incorporating ecosystem service trade-off/synergy into urban planning can support to design the effective urban planning in mitigating trade-off and promoting the overall benefits of urban ecosystem services (Feng et al. 2017; Gong et al. 2019). To our knowledge, however, few studies have examined the impacts of land cover change on multiple ecosystem services and their trade-off/synergy relationships. Moreover, emphasis has been placed on the quantitative analysis of the trade-off/synergy relationships over historical periods (Braun et al. 2018; Jia et al. 2014), and research on the consequences of future land cover change scenarios on ecosystem services considering different urban development strategies is lacking. Without conducting trade-off and synergy assessment, it remains unclear how these interactions vary spatiotemporally in the future under various land cover scenarios (Kragt and Robertson 2014).

With Nanjing as a case study, this study aims to assess the potential impacts of urban expansion on ecosystem services and their interactions in the future and to provide urban planning and ecosystem management guidance for sustainable urban development. The specific objectives of this study were to (1) reveal the change in ecosystem services during the urbanization process, (2) simulate various future land cover scenarios considering different urban development policies, (3) assess the potential effects of future urban land cover changes on ecosystem services and their trade-off/synergy relationships under different scenarios, and (4) propose urban development strategies for mitigating negative effects of land cover change on ecosystem services.

## Material and methods

### Study area and data sources

**Fig. 1:**
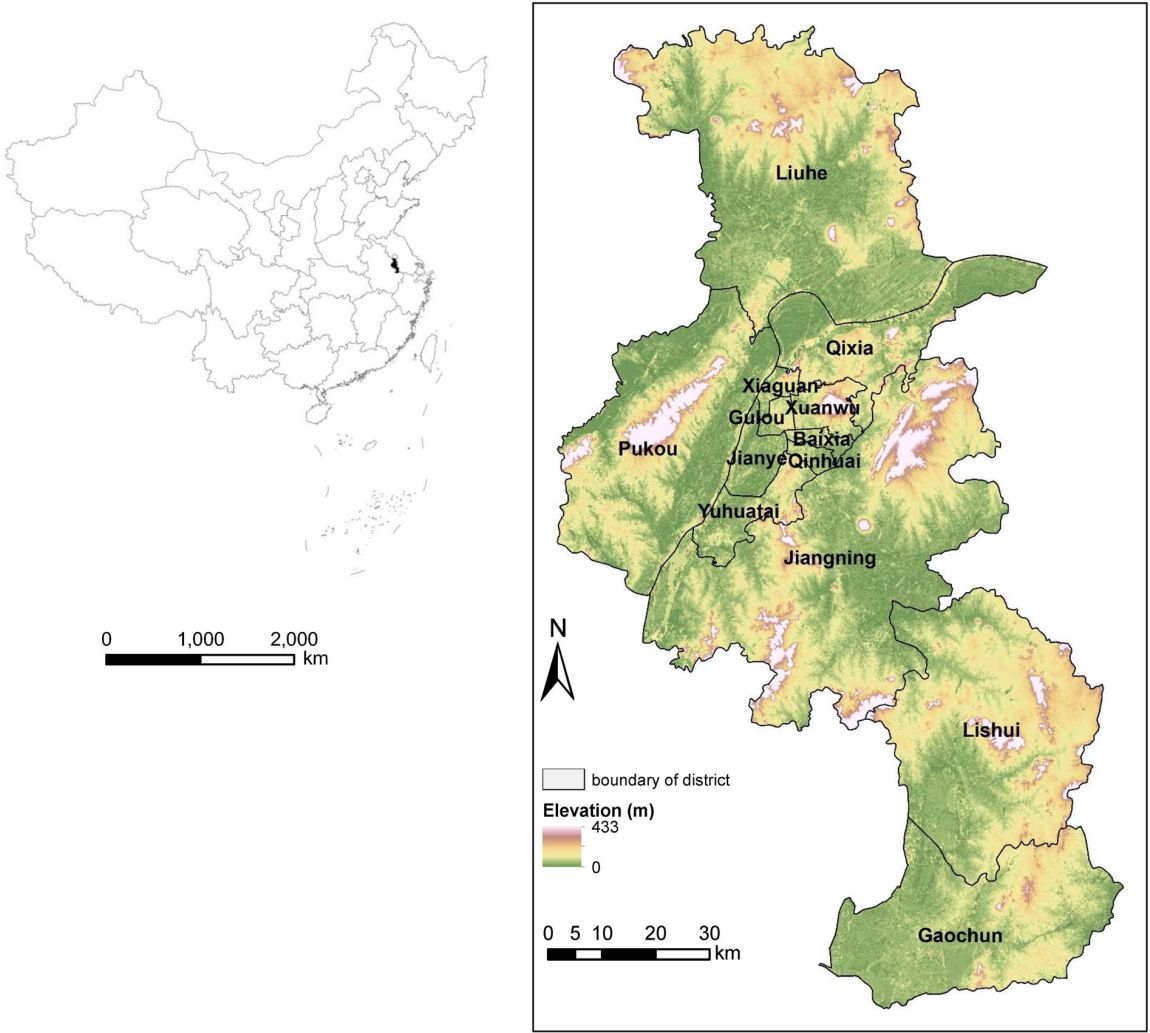
Location of the study area

The city of Nanjing lies in the eastern part of China (Fig. 1). Nanjing covers an administrative area of 6585 km^2^. It encompasses 12 municipal districts, city core districts (Xuanwu, Qinhuai, Gulou, Jianye, Xiaguan, Baixia), and suburban districts (Yuhuatai, Qixia, Pukou, Liuhe, Jiangning, Lishui, Gaochun). As one of the central cities in Yangtze River Delta region, Nanjing is recognized as one of the rapidly developing cities in China. The gross domestic product (GDP) of Nanjing notably increased from 241.1 to 972.1 billion RMB from 2005 to 2015. The total population in Nanjing was approximately 8.24 million in 2015 (Nanjing Statistical Bureau 2016). Due to the rapid development, some ecological problems have emerged that seriously threaten sustainable development and adversely influence human well-being. These problems include water quality degradation, regional carbon balance capacity decline, and air and soil pollution aggravation. These ecological problems impose a considerable negative impact on ecological security and pose challenges for realizing sustainable development in Nanjing. Like many other cities in the world, Nanjing faces the challenge of balancing the conflicting aspects of increasingly intensive human activities and ecological security.

Landsat 5 Thematic Mapper (TM) images of 2005 and 2010 and the Landsat 8 Operational Land Imager (OLI) image of 2015 were selected as the main data for deriving land cover maps, and these remote sensing images were obtained from the US Geological Survey (USGS). By adopting the maximum likelihood classification method, six land cover types (cropland, forest, grassland, wetland, built-up land, and bare land) were derived from Landsat images. To assess the classification accuracy, topographic and field survey data were collected and adopted as reference data. The calculated overall accuracies for 2005, 2010, and 2015 were 91.5%, 93.0%, and 92.5%, respectively. Soil property data were obtained from the Chinese Soil Data Set of the Harmonized World Soil Database (HWSD). Digital elevation model (DEM) data with a spatial resolution of 30 m were obtained from the USGS. Meteorological data of Nanjing city, including monthly and annual precipitation, monthly temperature, and annual accumulated temperature, were acquired from the National Meteorological Information Center of China. In addition, a set of ancillary data, including a road map, location of the city center, and population data, were obtained from the Nanjing Bureau of Planning and Natural Resources.

### Ecosystem service valuation

Considering the importance of ecosystem services and data availability in Nanjing city, six ecosystem service types were selected for assessment and analysis, including water yield (WY), carbon storage (CS), soil conservation (SC), air purification (AP), crop production (CP), and habitat quality (HQ).

The InVEST model quantifies the relative contributions of water from different parts of a landscape, thus providing insight into how changes in land use patterns influence the annual surface WY. The annual WY (WY_*x*_) value for pixel *x* is calculated with the following equation:

1 $$ {\mathrm{WY}}_x=\left(1-{\mathrm{AET}}_x/{p}_x\right)\times {p}_x $$

where *p*_*x*_ is the average annual precipitation value for pixel *x* and AET_*x*_ is the evapotranspiration value for pixel *x*. The biophysical value for different land cover type was presented in Table A.1.

The CS amount was calculated with the CS and sequestration module in the InVEST model based on land use maps and four carbon pools, including the aboveground and belowground biomasses and soil and dead organic matter. The CS amount can be estimated with the following equation:

2 $$ \mathrm{CS}={\sum}_{lp=1}^6{\mathrm{SA}}_{lp}\times \left({\mathrm{CD}}_{lp}^{\mathrm{above}}+{CD}_{lp}^{\mathrm{below}}+{\mathrm{CD}}_{lp}^{\mathrm{soil}}+{\mathrm{CD}}_{lp}^{\mathrm{dead}}\right) $$

where SA_*lp*_ is the area of land use type *lp* and ${\mathrm{CD}}_{lp}^{\mathrm{above}}$, ${\mathrm{CD}}_{lp}^{\mathrm{below}}$, ${\mathrm{CD}}_{lp}^{\mathrm{soil}}$, and ${\mathrm{CD}}_{lp}^{\mathrm{dead}}$ are the carbon densities of the different carbon pools for land use type *lp*. The biomass carbon density for the different land use types was derived from the results of a relevant study (Chuai et al. 2013) (Table A.2).

In this study, the sediment retention module in the InVEST model was applied to estimate the SC amount in the Yangtze River Delta (YRD) region. The module adopts the Revised Universal Soil Loss Equation (RUSLE). The SC amount SC_*x*_ for pixel *x* can be calculated with the following equation:

3 $$ {\mathrm{SC}}_x={\mathrm{Ap}}_x-{\mathrm{Ar}}_x $$

where Ap_*x*_ is the amount of potential soil erosion and Ar_*x*_ is the amount of actual soil erosion. Ap_*x*_ and Ar_*x*_ can be estimated as follows:

4 $$ {\mathrm{Ap}}_x={R}_x\times {K}_x\times {L}_x\times {S}_x $$

5 $$ {\mathrm{Ar}}_x={R}_x\times {K}_x\times {L}_x\times {S}_x\times {C}_x\times {P}_x $$

where *R*_*x*_ is the rainfall erosion factor for pixel *x*, *K*_*x*_ is the soil erosion factor, *L*_*x*_ is the slope length factor, *S*_*x*_ is the slope, *C*_*x*_ is the vegetation cover factor, and *P*_*x*_ is the support practice factor. In this study, *C*_*x*_ and *P*_*x*_ were identified based on a previous relevant study (Qiao et al. 2019) (Table A.3).

Previous studies have proven that the normalized difference vegetation index (NDVI) value for cropland pixels exhibits a significant linear correlation with crop production. Therefore, the crop production value for each pixel was calculated based on the NDVI value and related district-level statistical data on crop production, following Peng et al. (2016).

6 $$ {\mathrm{CP}}_{x,i}=\left({\mathrm{NDVI}}_{x,i}/{\mathrm{NDVI}}_{\mathrm{sum},i}\right)\times {\mathrm{Crop}}_i $$

where CP_*x*, *i*_ represents the crop production value for cropland pixel *x* in district *i*; NDVI_*x*, *i*_ and NDVI_sum, *i*_ are the maximum NDVI value for cropland pixel *x* in district *i* and the total NDVI value for district *i*, respectively; and Crop_*i*_ represents the crop production value for district *i*.

Air pollution can significantly influence the health of humans. The inhalable particulate matter with a diameter generally 10 μm and smaller (PM10) is the most important and well-documented pollutant in the YRD region. It is acknowledged that vegetation can remove PM10 from the atmosphere and improve the air quality. The air purification (AP) for Nanjing is quantified using the following equation proposed by Landuyt et al. (2016):

7 $$ \mathrm{AP}={\sum}_{lp=1}^6{A}_{lp}\times {\mathrm{PM}}_{lp} $$

where *A*_*lp*_ is the area of land use type *lp* and PM_*lp*_ is the amount of removed PM10 per unit area of land use type *lp*. The PM_*lp*_ values for the different land use types are determined according to relevant study (Xie et al. 2018) (Table A.4).

The HQ of Nanjing was measured using Habitat Quality module from InVEST model. The HQ value for pixel *x* is calculated by considering the intensity of external stress from threats and the relative suitability of each land cover type to each threat. The HQ value can be expressed as

8 $$ {\mathrm{HQ}}_{xk}={H}_k\times \left(1-{D}_{xk}^z/\left({D}_{xk}^z+{S}^z\right)\right) $$

where HQ_*xk*_ refers to the HQ value for pixel x with land cover type k. *H*_*k*_ is the habitat suitability score for land cover type *k*. ${D}_{xk}^z$ indicates the total threat level for pixel x with land cover type k. *S* is the half-saturation constant. *z* is a normalized constant (Xie et al. 2018) (Table A.5).

To analyze the trade-off/synergy relationships among the multiple ecosystem services, an innovative trade-off/synergy degree (TSD) index was developed and adopted to quantify the magnitude of the complex interactions. The TSD index for ecosystem services *i* and *j* (TSD_*i*, *j*_) over the periods of *t*2 and *t*1 was calculated with Eq. (8):

9 $$ {\mathrm{TSD}}_{i,j}^{t2-t1}=\left\{\begin{array}{c}\kern0.5em 0\kern2.75em \left({\Delta  \mathrm{ES}}_i^{t2-t1}\times {\Delta  \mathrm{ES}}_j^{t2-t1}=0\right)\\ {}\kern0.5em \left|\ln \left|{\Delta  \mathrm{ES}}_i^{t2-t1}/{\Delta  \mathrm{ES}}_j^{t2-t1}\right|\right|\kern0.5em \left({\Delta  \mathrm{ES}}_i^{t2-t1}\times {\Delta  \mathrm{ES}}_j^{t2-t1}>0\right)\ \\ {}\begin{array}{cc}-\left|\ln \left|{\Delta  \mathrm{ES}}_i^{t2-t1}/{\Delta  \mathrm{ES}}_j^{t2-t1}\right|\right|& \left({\Delta  \mathrm{ES}}_i^{t2-t1}\times {\Delta  \mathrm{ES}}_j^{t2-t1}<0\right)\end{array}\ \end{array}\right. $$

where ${\Delta  \mathrm{ES}}_i^{t2-t1}$ and ${\Delta  \mathrm{ES}}_j^{t2-t1}$ are the relative changes in ecosystem services *i* and *j* over the period of *t*2–*t*1, which are calculated as follows:

10 $$ \left\{\begin{array}{c}{\Delta  \mathrm{ES}}_i^{t2-t1}=\left({\mathrm{ES}}_i^{t2}-{\mathrm{ES}}_i^{t1}\right)/{\mathrm{ES}}_i^{t1}\\ {}{\Delta  \mathrm{ES}}_j^{t2-t1}=\left({\mathrm{ES}}_i^{t2}-{\mathrm{ES}}_j^{t1}\right)/{\mathrm{ES}}_j^{t1}\end{array}\right. $$

where ${\mathrm{ES}}_i^{t2}$ and ${\mathrm{ES}}_i^{t1}$ are the values of ecosystem service *i* at time points *t*2 and *t*1, respectively. According to Eq. (8), if ${\Delta  \mathrm{ES}}_i^{t2-t1}\times {\Delta  \mathrm{ES}}_j^{t2-t1}=0$, ${\mathrm{TSD}}_{i,j}^{t2-t1}$=0, suggesting that no synergy or trade-off relationship exists between ES_*i*_ and ES_*j*_. If ${\Delta  \mathrm{ES}}_i^{t2-t1}\times {\Delta  \mathrm{ES}}_j^{t2-t1}>0$, there exists a synergy relationship between ES_*i*_ and ES_*j*_, and the level of synergy is $\left|\ln \left|{\Delta  \mathrm{ES}}_i^{t2-t1}/{\Delta  \mathrm{ES}}_j^{t2-t1}\right|\right|$. If ${\Delta  \mathrm{ES}}_i^{t2-t1}\times {\Delta  \mathrm{ES}}_j^{t2-t1}<0$, a trade-off relationship exists between two ecosystem services, and the level of trade-off can be represented by $-\left|\ln \left|{\Delta  \mathrm{ES}}_i^{t2-t1}/{\Delta  \mathrm{ES}}_j^{t2-t1}\right|\right|$.

### Development of the land cover change model

The Markov-cellular automata (CA) model was developed to support decision making related to sustainable ecosystem management by simulating alternative scenarios. The model is composed of three components, including a Markov chain (MC), logistic regression (LR), and CA. The simulation of future land cover change is conducted in three steps: (1) MC analysis is conducted to derive future land cover transition matrices based on the land cover maps of 2005, 2010, and 2015; (2) the LR model is adopted to generate a transition potential map for each land cover type; and (3) the CA model is implemented to predict the spatial distribution of the various land cover types according to the transition matrices and transition potential maps.

The MC model is commonly adopted to predict the probability of change from one land cover type to another based on the historical transition probability matrix derived from the two former land cover maps (Halmy et al. 2015). The transition probability matrix for the target simulation period can be obtained with the following equation:

11 $$ {\mathrm{S}}_{t+1}={\mathrm{S}}_t\times {\mathrm{TPM}}_{ij} $$

where *S*_*t* + 1_ and *S*_*t*_ are the land cover states at time points *t* + 1 and *t*, respectively, and TPM_*ij*_ is the transition probability matrix expressed as follows:

12 $$ {\mathrm{TPM}}_{ij}=\left[\begin{array}{cccc}{P}_{11}& {P}_{12}& \dots & {P}_{1n}\\ {}{P}_{21}& {P}_{22}& \dots & {P}_{2n}\\ {}\dots & \dots & \dots & \dots \\ {}{P}_{n1}& {P}_{n2}& \dots & {P}_{nn}\end{array}\right] $$

where *P*_*ij*_ is the transition probability from land cover type *i* to land cover type *j* from time point *t* to time point *t* + 1 and *n* is the number of land cover types.

The land cover maps of 2005 and 2015 for Nanjing city were first adopted to project the transition probability matrices of the land cover types between 2005 and 2015. An MC model assumes that the transition probability of land cover types at the next stage depends only on the current probability (Pijanowski et al. 2002). Therefore, the matrices were further applied to predict the transition probability over the future period. The MC model controls the future transitions among the different land cover types based on the derived transition matrices.

Given that the MC model only calculates the transition probabilities and areas among the various land cover types, the spatial distribution of the transitions is inadequately involved (Zhao et al. 2019). The CA model was developed and is implemented in this study to simulate the spatiotemporal evolution of land cover dynamics.

The state of cell *x* at time *t* + 1 (*S*_*x*, *t* + 1_) can be calculated as a function of the state of the cell at time *t*, the state of the neighborhood of cell *x* (Ω_*x*, *t*_), and the transition potential value (*TP*_*x*, *t*_) in the CA model. A 5 × 5 contiguity filter was selected as the neighborhood definition.

13 $$ {S}_{x,t+1}=f\left({S}_{x,t},{\Omega}_{x,t},{\mathrm{TP}}_{x,t}\right) $$

The transition potential values determine the probability that the cells of a certain land cover type will be changed to another land cover type. The transition potential value TP_*x*_ for cell *x* can be calculated based on the LR model:

14 $$ {\mathrm{TP}}_x=\exp (z)/\left[1+\exp (z)\right] $$

15 $$ \mathrm{z}={b}_0+{b}_1{x}_1+{b}_2{x}_2+\dots +{b}_n{x}_n $$

where *x*_*i*_(*i* = 1, …, *n*) is the potential suitability factor. A series of variables that contribute to land cover change are involved, which include the slope, distance to a major road (D2MajR), distance to a minor road (D2MinR), distance to the city center (D2Cen), and population density (PopDen). *b*_0_ is the intercept, and *b*_*i*_(*i* = 1…*n*) are the corresponding weights of all factors.

When the TP_*x*_ value for cell *x* is derived, the CA model can identify the cells of one land cover type that are converted into another type and the cells that remain unchanged. The iteration steps of reallocating land cover stop when the total land demand predicted by the MC model is satisfied. We first simulated the land cover map of 2015 based on the actual land cover maps of 2005 and 2010. Compared with the actual land cover map of 2015, the overall accuracy and Kappa index were 92% and 0.83, respectively.

### Simulation of the future scenarios

The Markov-CA model was adopted to simulate alternative land cover change scenarios for Nanjing in 2030. Based on the historical land cover change trend and future development goals, three scenarios were designed: business-as-usual (BAU), cropland protection (CP), and ecological restoration (ER) scenarios.

The BAU scenario strictly follows the historical trend of the land cover change without any limitations or alterations. Due to the imbalance between the limited cropland and high crop production demand, the central government of China has announced a goal of ensuring that a minimum of 1.8 billion mu (121 million hectares) of land must be used for farming purposes. By the end of 2016, however, China had a total of 134.95 million hectares of farmland, down 0.04 million hectares from the 2015 level. In recent decades, rapid urbanization has posed an enormous challenge to the stability and sustainability of food production in China. In this study, therefore, a CP scenario was designed to protect the cropland being developed and to transform grassland and forestland with slopes smaller than 6° into cropland. The grassland and forestland in the city core districts will not be involved in the transformation. Considering the potential effect of ER policies on the ecology, we designed an ER scenario, which highlights ecological protection and avoids the environmental problems caused by human activities. To mitigate the negative effects of human activities on ecology, constraints were involved in the simulation model to prevent ecologically sensitive areas from being cultivated or urbanized.

## Results

### Land use change in Nanjing from 2005 to 2015

**Fig. 2:**
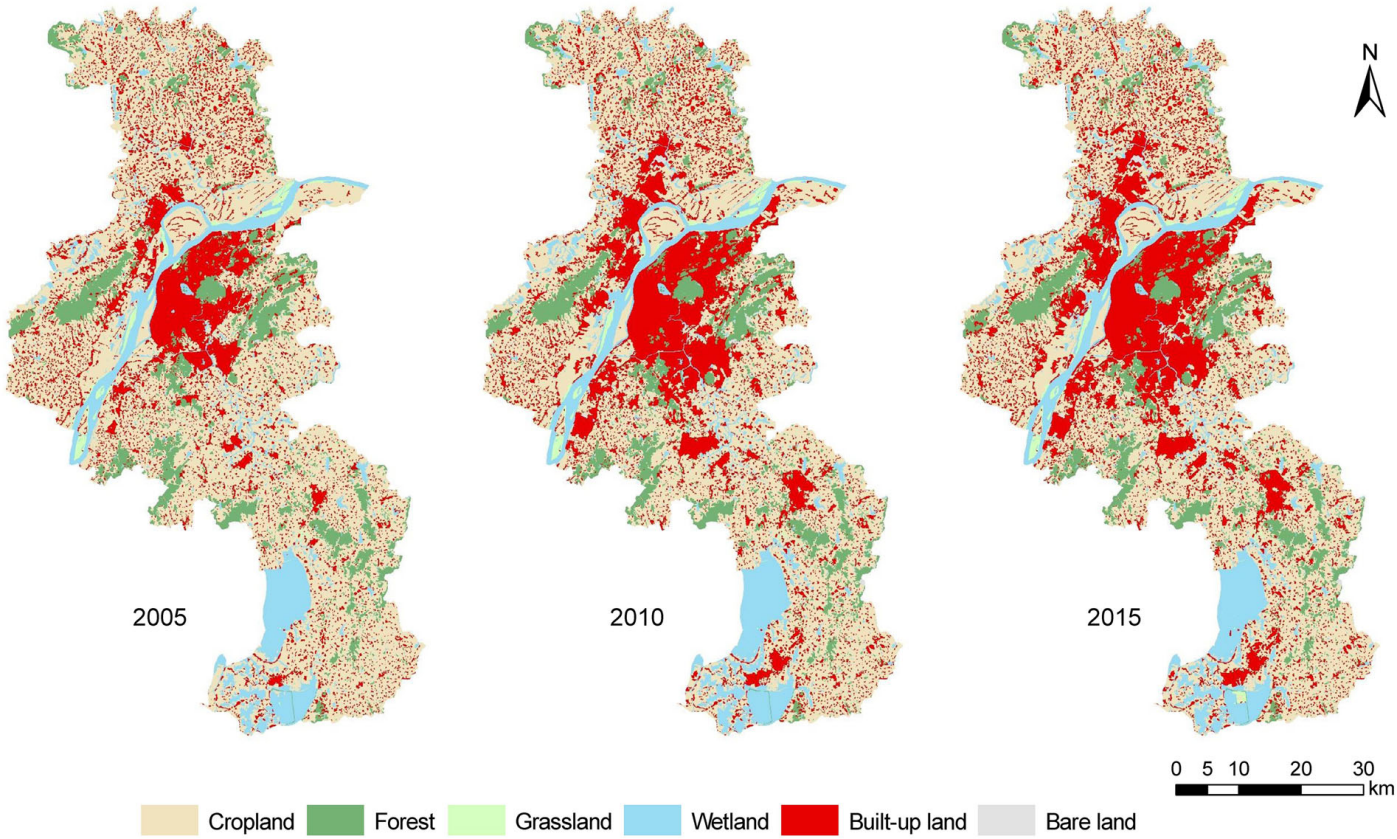
Land cover maps for 2005, 2010, and 2015

Figure 2 presents the land cover maps obtained from Landsat images of 2005, 2010, and 2015. The main land cover types in Nanjing city were cropland, forest, wetland, and built-up land, which accounted for 52.17%, 10.16%, 11.46%, and 24.92%, respectively, of all land cover types (Table 1). Nanjing city experienced dramatic land cover changes over the period of 2005–2015. Among the six land cover classes, built-up land increased the most, while cropland decreased the most, followed by forestland.

**Table 1:** The statistics of land cover over time in terms of area and percentage of each type

Land cover type	2005		2010		2015		2005–2010	2010–2015	2005–2015
	km^2^	%	km^2^	%	km^2^	%	km^2^	km^2^	km^2^
Cropland	3944.37	59.93	3509.21	53.31	3433.98	52.17	− 435.1	− 75.23	− 510.39
Forest	696.04	10.57	673.56	10.23	668.57	10.16	− 22.48	− 4.99	− 27.47
Grassland	59.64	0.91	54.20	0.82	57.84	0.88	− 5.44	3.64	− 1.80
Wetland	716.82	10.89	765.88	11.64	754.57	11.46	49.06	− 11.31	37.75
Built-up land	1161.26	16.04	1552.16	23.58	1640.29	24.92	390.90	88.13	479.03
Bare land	3.95	0.06	27.07	0.41	26.83	0.41	23.12	− 0.24	22.88

Nanjing was subject to dramatic urban expansion, with built-up land increasing 41.25%, from 1161.26 km^2^ in 2005 to 1640.29 km^2^ in 2015. Furthermore, it is evident that the annual increase rate of built-up land declined over the two different periods. Cropland decreased from 3944.37 km^2^ (59.93% of the total area) in 2005 to 3433.98 km^2^ (52.17% of the total area) in 2015. Specifically, cropland decreased from 2005 to 2010, accounting for 85.25% of the total decreasing cropland area. Due to the rapid urbanization process, forestland gradually decreased 27.47 km^2^ over 10 years. Wetland increased 49.06 km^2^ from 2005 to 2010 and decreased 11.31 km^2^ from 2010 to 2015.

A total of 479.03 km^2^ of land was changed into built-up land, representing 7.28% of the total area of Nanjing. Built-up land increased 436.23 km^2^ from the conversion of cropland, and this value represented 91.07% of the newly developed built-up land. Specifically, built-up land received 393.72 and 42.51 km^2^ from cropland over the periods of 2005–2010 and 2010–2015, respectively. It highlights the serious conflict between the limited land resources and the very high demand for urban development.

### Spatiotemporal dynamics of ecosystem services from 2005 to 2015

**Fig. 3:**
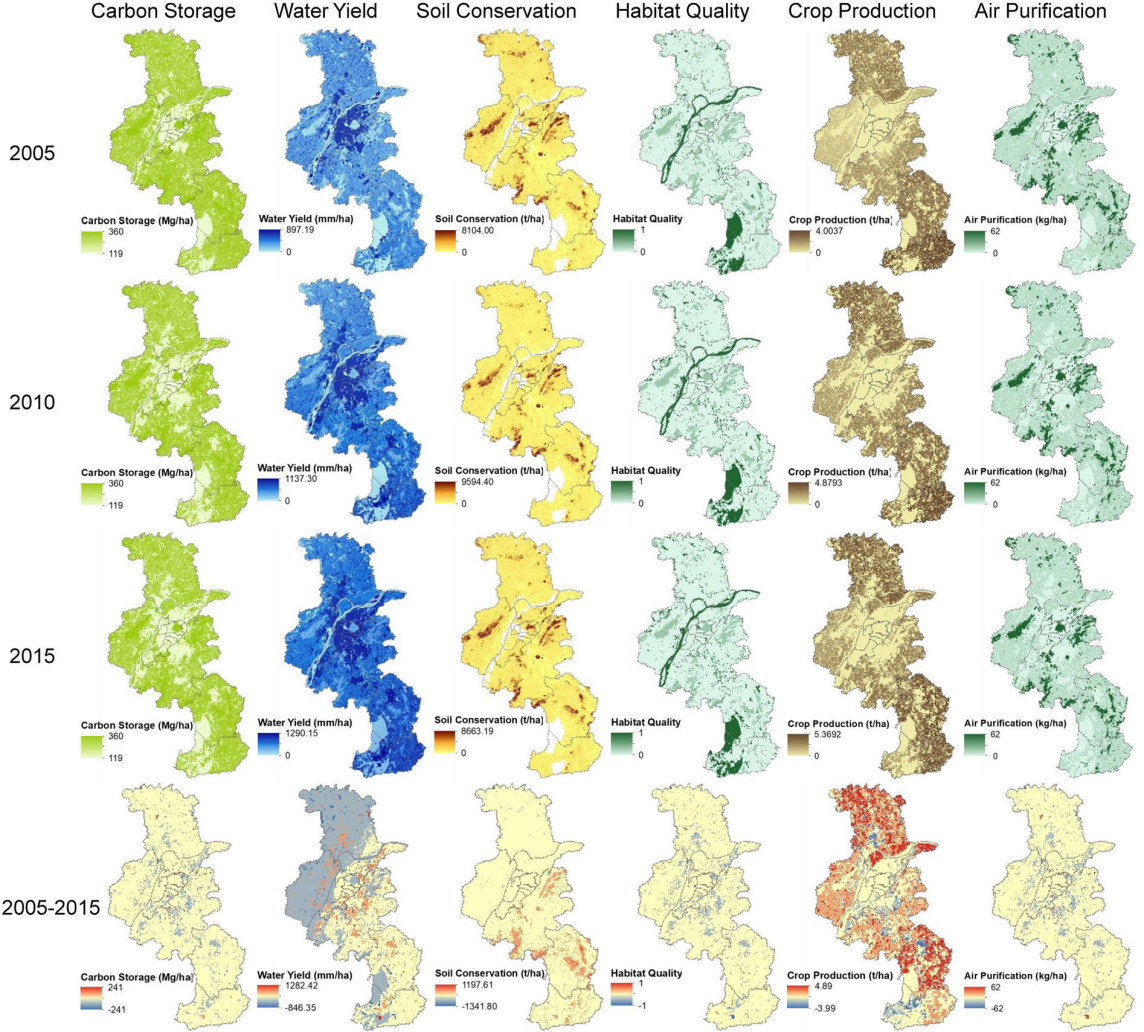
Spatial distributions of multiple ecosystem services for 2005 (first row), 2010 (second row), 2015 (third row), and change of ecosystem services from 2005 to 2015 (forth row)

Figure 3 shows the spatial distribution of the ecosystem services over the period of 2005–2015. Over the past 10 years, the total values of CS, HQ, and AP in Nanjing declined 3.05%, 5.86%, and 7.97%, respectively. WY increased from 1.59 ∗ 10^15^ in 2005 to 2.94 ∗ 10^15^ in 2015 (Table 2). SC increased from 2005 to 2010 and then declined from 2010 to 2015, mainly as a result of precipitation variation. Crop production gradually increased from 9.65 ∗ 10^5^ to 11.41 ∗ 10^5^ t from 2005 to 2015.

**Table 2:** The values of various ecosystem services from 2005 to 2015 in Nanjing city

	2005	2010	2015	2005–2010	2010–2015	2005–2015
Carbon storage (10^8^ t)	1.37	1.34	1.33	− 2.19%	− 0.75%	− 2.92%
Habitat quality (10^5^)	2.24	2.15	2.11	− 4.02%	− 1.86%	− 5.80%
Water yield (10^15^ t)	1.59	2.21	2.94	38.99%	33.03%	84.91%
Soil conservation (10^7^ t)	1.18	1.29	1.19	9.32%	− 7.75%	0.85%
Crop production (10^5^ t)	9.65	11.06	11.41	14.61%	3.16%	18.24%
Air purification (10^3^ t)	8.09	7.54	7.45	− 6.80%	− 1.19%	− 7.91%

The total CS amount was 1.37 ∗ 10^8^ t in 2005, decreasing to 1.34 ∗ 10^8^ t in 2010 and to 1.33 ∗ 10^8^ t in 2015. In terms of the spatial distribution of the ecosystem services, the low CS values were concentrated in the city core districts, especially in Gulou, Qinhuai, and Baixia districts. The surrounding suburban districts, such as Jiangning and Lishui districts, performed better in terms of CS, which can be attributed to the higher proportion of forestland and cropland in the suburban districts of Nanjing. During the study period, the highest decrease rate in Nanjing city occurred in Yuhuatai district, with a decrease rate of 8.93%, which is mainly due to the rapid urban expansion of this district. Baixia district exhibited the most notable increase in CS from 2005 to 2015.

Regarding HQ, Gaochun and Lishui districts attained higher values over the period of 2005–2015. The HQ values in the surrounding suburban districts were higher than that in the city core. Along with the urbanization process in Nanjing, the HQ values decreased in all districts except Baixia district. The highest HQ decrease rate per unit area was found in Yuhuatai district, which followed a similar trend to that of CS. This can be explained by the fact that the proportion of vegetation coverage increased during the study period.

The WY value in Nanjing was 1.59 ∗ 10^15^ t in 2005. In 2010, the WY value was 2.21 ∗ 10^15^ t, with a total value of 0.62 ∗ 10^15^ t and a 38.99% increase over the 2005 level. The total value increased to 2.94 ∗ 10^15^ t in 2015, with a total value increase of 33.03% over the 2010 level. The high WY values per unit area in Nanjing were mainly located in the city core, such as Gulou, Qinhuai, and Baixia districts, while the lower WY values per unit area were concentrated in the suburban districts. This can be explained by the fact that the lower vegetation coverage in the city core resulted in a lower evapotranspiration value. The lower WY value per unit area was attributed to the higher vegetation coverage in the suburban districts.

Over the first period (2005–2010), the total SC value increased from 1.18 ∗ 10^7^ to 1.29 ∗ 10^7^ t, with an overall total of 0.11 ∗ 10^7^ t and a 9.32% increase from 2005 to 2010. The total SC value in Nanjing was 1.19 ∗ 10^7^ t in 2015, an overall total increase of 0.85% over the 2005 level. The higher SC values were mainly distributed in Xuanwu and Jiangning districts. The lower SC values were concentrated in the city core, such as Qinhuai and Baixia districts. This mainly occurs because the steeper slope and higher precipitation resulted in higher rainfall erosion and soil loss.

The crop production value had the similar change trend from 2005 to 2015 but exhibited distinct regional differences. The mean crop production value in Liuhe, Lishui, and Gaochun districts was higher than that in the other districts. The crop production decrease rates in Jianye, Yuhuatai, and Xuanwu districts were higher than 40%. As indicated by the reduction of crop production in the city core, the rapid urban expansion in Nanjing led to a major loss in cropland, with a great effect on the crop production value. However, suburban districts such as Pukou and Liuhe attained increasing crop production values higher than 40%. Furthermore, the high crop production values were located in the suburban districts, especially Lishui, Gaochun, and Liuhe districts. Although cropland decreased 12.94% in Nanjing city over the period of 2005–2015, the crop production values increased in all suburban districts due to the implementation of improved agricultural technologies. The crop production per unit area was thus improved.

Moreover, the AP value, measured by the captured PM_10_, decreased from 8.09 ∗ 10^3^ t in 2005 to 7.45 ∗ 10^3^ t in 2015, at a reduction rate of 7.91%. Over the first study period (2005–2010), the decrease rate was higher than that over the last period (2010–2015). The mean AP value in Xuanwu district was the highest. There were lower mean AP values in the city core districts such as Gulou, Qinhuai, and Baixia. Additionally, Baixia district attained a notable increase, and the AP growth rate per unit area was 291.67%. Notable decreases in the mean AP value occurred in Qinhuai district, at a decrease rate of 61.56%. The rapid land cover change and loss of forestland and cropland led to the high AP decrease rate.

### Land cover change under the different scenarios in the future

**Fig. 4:**
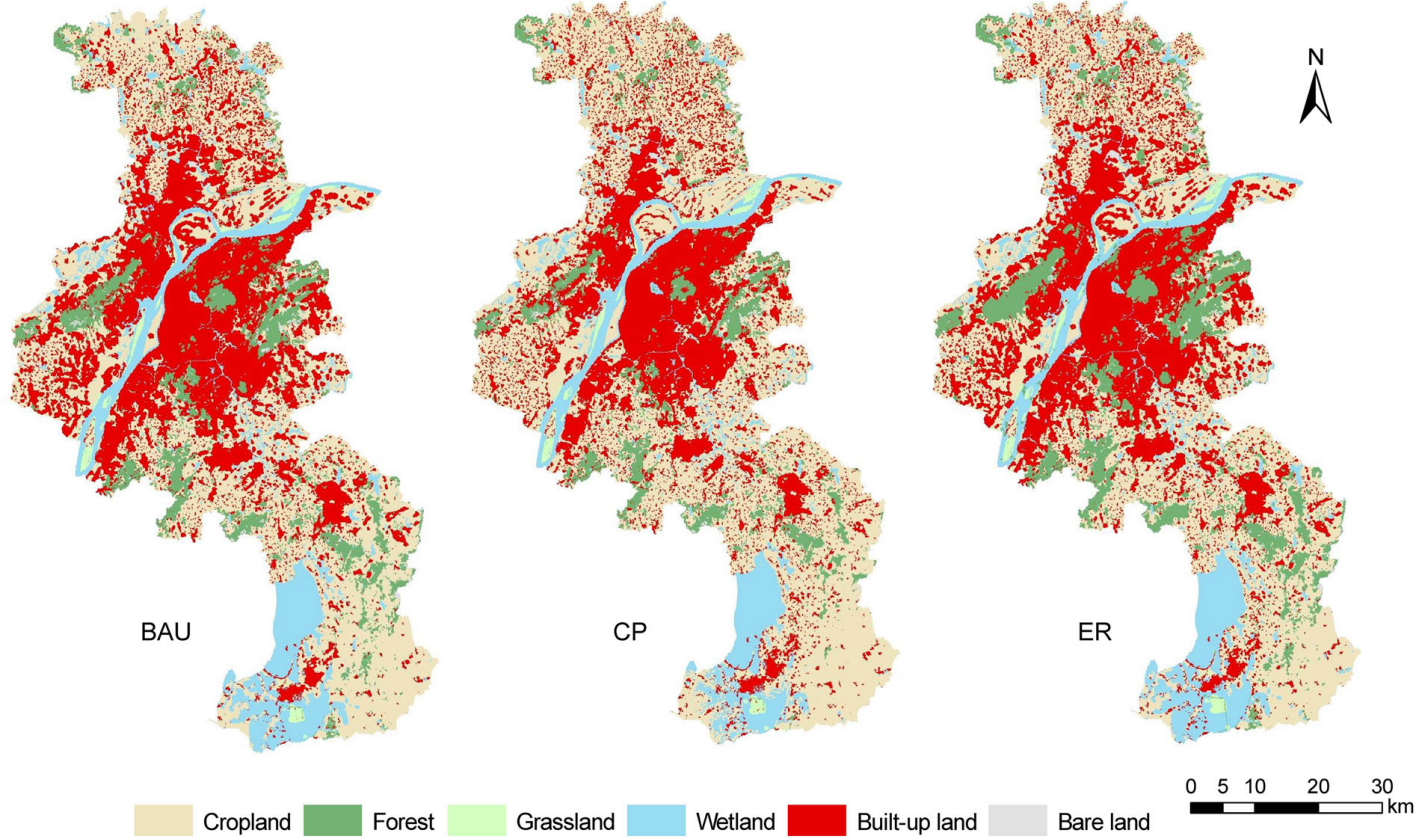
Simulated land cover maps in 2030 under business as usual (BAU), crop protection (CP), and ecological restoration (ER) scenarios

By applying the Markov-CA model, the design modeling configuration for 2010–2015, and the land cover data of 2015, different development scenarios were generated to simulate alternative spatial patterns of the land cover in Nanjing by 2030 (Fig. 4). The BAU scenario was simulated on the basis of assumption that the trend of land cover change would continue without any change. The land cover change trend over the 2015–2030 period was consistent with that over the 2010–2015 period. Under the BAU scenario, the built-up land in Nanjing is projected to increase 33.81%, from 1640.29 km^2^ in 2015 to 2194.80 km^2^ in 2030. Moreover, compared with the land cover map of 2015, cropland decreased 17.52%, and forestland decreased 3.94%.

The ER scenario highlights the protection of environmentally sensitive areas. Some regions in the city core kept unchanged because of the environmental constraints. Notably, the majority of the newly developed built-up land was transformed from cropland. Under the ER scenario, built-up land is expected to increase 26.51% over the period of 2015–2030. As evidenced by the 18.19% decrease in cropland, more cropland will be occupied by newly developed built-up land. In contrast to the BAU and CP scenarios, forestland and grassland exhibited major increases. Forestland increased 17.30% due to the implementation of strict ecological protection policies.

The CP scenario can be characterized by the strict protection of cropland. Newly developed built-up land is more likely to be converted from forestland and grassland. In addition, forestland with a slope smaller than 6° was transformed into cropland. The growth of built-up land in Nanjing will be 204.86 km^2^, representing a 12.49% increase over the 2015 level. Under the CP scenario, cropland will be strictly protected from being developed. Therefore, the area of suitable land for urbanization will be smaller than that under the other scenarios. Furthermore, cropland will decrease 0.35% from 2015 to 2030. However, more forestland and grassland will be occupied by built-up land.

### Potential impacts of land cover change on ecosystem services

**Fig. 5:**
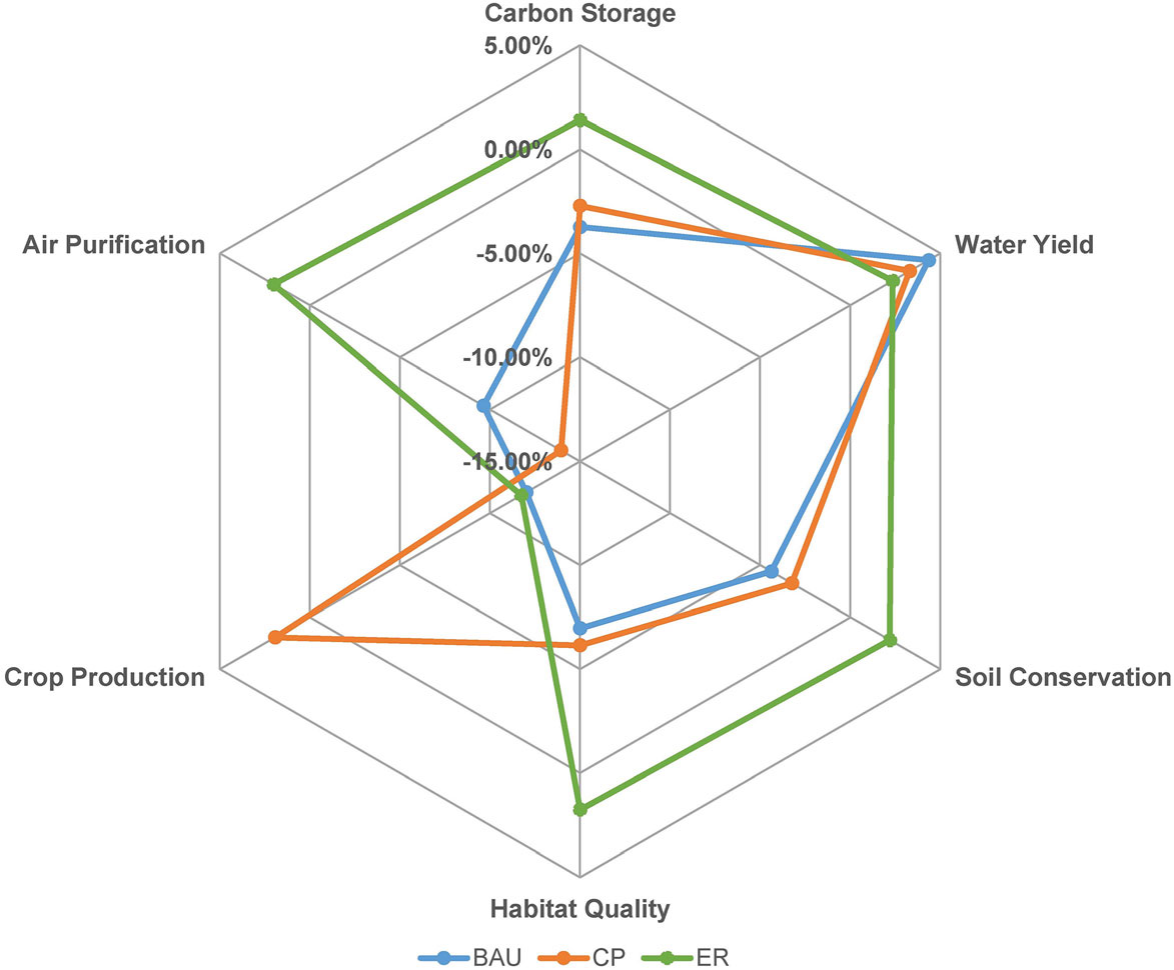
Change ratios of multiple ecosystem services under three scenarios compared with the year of 2015

Under the BAU scenario in 2030, compared with the ecosystem services in 2015, CS, SC, HQ, crop production, and air regulation will decrease 3.73%, 4.38%, 6.97%, 12.01%, and 9.63%, respectively, as shown in Fig. 5. The decline in ecosystem services is mainly due to the major transformation from cropland and vegetated land into built-up land from 2015 to 2030 under the BAU scenario. The WY value in Nanjing exhibits the most notable increase trend from 2015 to 2030, with the WY value increasing 4.35%. WY has a trade-off relationship with the other ecosystem services. This occurs because the rapid expansion of built-up land occupies a large amount of cropland and vegetated land, which have higher ecosystem service values. The increasing area of built-up land enhances WY because of its low evaporation capability.

Under the CP scenario, trade-off relationships exist between crop production and regulating ecosystem services, including CS, HQ, AP, and SC. The increase in crop production can lead to a decrease in these regulating ecosystem services. Notably, the crop production value will increase 1.93%, from 11.41 ∗ 10^5^ to 11.63 ∗ 10^5^ t over 15 years under the CP scenario. This is 15.84% and 15.49% higher than that under the BAU and ER scenarios, respectively. The AP value will decrease 13.95% from 2015 to 2030 under the CP scenario, thereby exhibiting the largest decrease among the three scenarios. This can be attributed to the transformation of vegetated land into cropland under the CP scenario.

Under the ER scenario, there exists a synergy relationship among all ecosystem services except crop production. The high ecosystem service values in Nanjing is accounted for by the notable loss in crop production. Crop production is projected to reach 10.07 ∗ 10^5^ t, representing an 11.74% decrease over the 2015 level. CS will increase 1.40% from 1.33 ∗ 10^8^ tons in 2015 to 1.35 ∗ 10^8^ tons in 2030 under the ER scenario, but it will notably decrease 3.73% under the BAU scenario and 2.70 under the CP scenario. SC in Nanjing will increase 2.19% but decrease 4.38% and 3.24% under the BAU and ER scenarios, respectively. Due to the strict implementation of ecology protection policies and the transformation of cropland with a slope larger than 6° into vegetated land, HQ will increase 1.76% from 2015 to 2030 under the ER scenario. The ER scenario performs better than the other two scenarios in terms of all ecosystem services except for crop production.

**Fig. 6:**
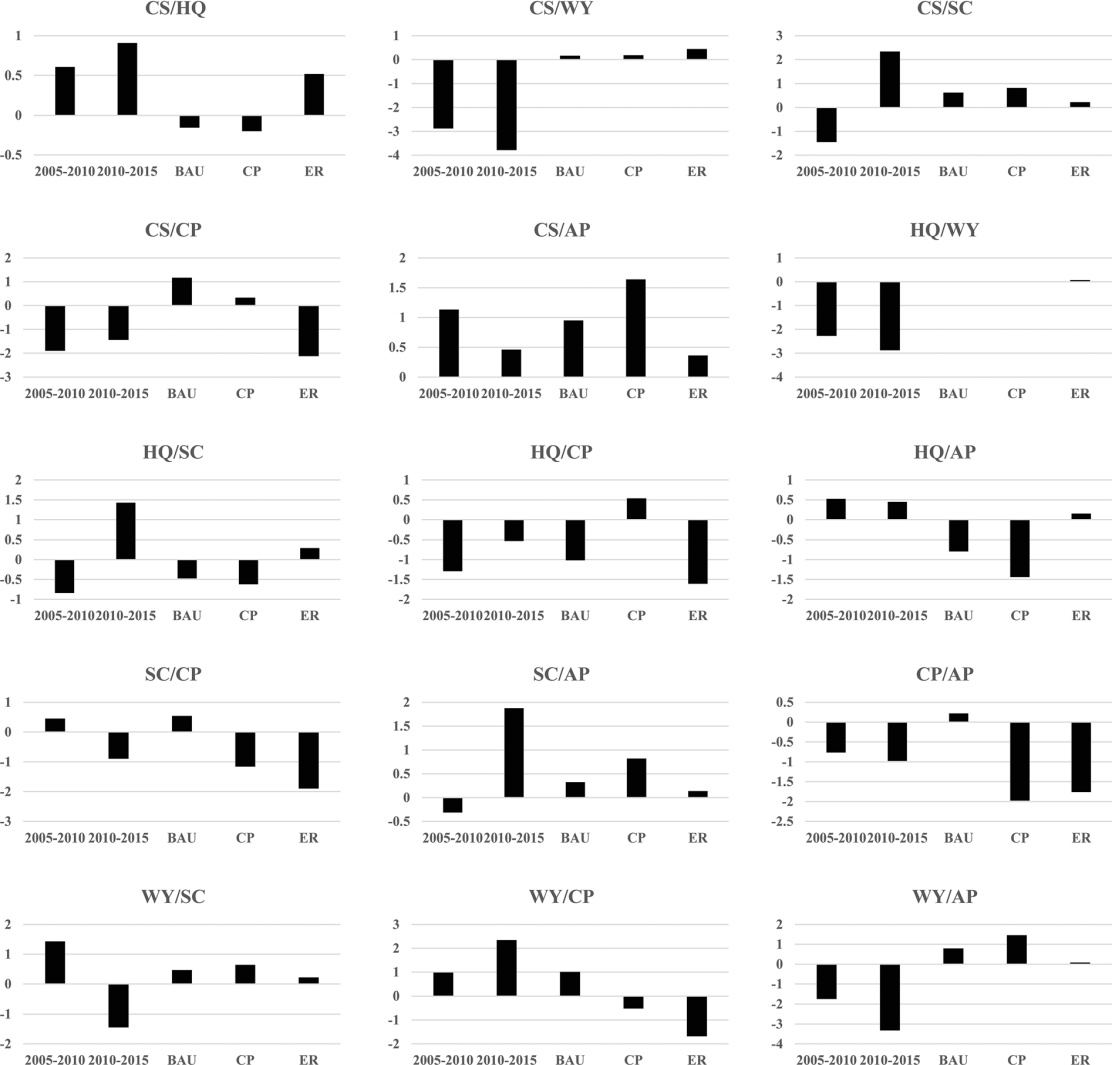
The degree of trade-offs and synergies revealed by TSD index for various pairs of ecosystem services. Note that CS, WY, SC, HQ, CP, and AP refer to carbon storage, water yield, soil conservation, habitat quality, crop production, and air purification, respectively

The TSD index was further adopted to represent the degree of trade-offs and synergies between two ecosystem services. As shown in Fig. 6, the TSD index value varies notably from 2005 to 2030 under the different scenarios. The relationship between CS and AP under the CP scenario exhibits the most notable synergy degree with a TSD index value of 1.64. The highest trade-off degree with a TSD index value of − 2.12 is found between CS and crop production under the ER scenario.

A change in urban development strategy could reverse the interaction relationship between ecosystem services. Compared with the BAU and CP scenarios, the relationship between CS and HQ was converted to a synergy relationship under the ER scenario. Compared with the BAU and ER scenarios, the strict implementation of CP policies could convert the trade-off relationship between HQ and crop production to a synergy relationship. Because of the implementation of CP policies, the relationship between crop production and HQ changes from a trade-off to a synergy under the CP scenario.

In addition, the variation in urban development policies could affect the trade-off and synergy degrees. Compared with the BAU and ER scenarios, the CP scenario transformed the interaction relationship between SC and AP from a nonsignificant synergy relationship to a significant synergy relationship. Compared with the BAU and CP scenarios, the trade-off magnitude between SC and crop production increased notably under the ER scenario. The degree of synergy between CS and AP notably increased under the CP scenario. The trade-off degree between SC and crop protection weakened when the development policy changed from ER to CP.

## Discussion

### Linking the Markov-CA model to ecosystem service models

Land cover change and the corresponding variation in ecosystem services have been a research area of increased interest for several years. Many previous studies have made efforts to assess the effects of land cover change on ecosystem services (Li and Wang 2018; Vo et al. 2015). To better understand the effects of land cover change on ecosystem services and to optimize the future development strategy in Nanjing, this study proposed a framework linking the Markov-CA model to ecosystem service models.

First, this model can involve different types of variables, such as environmental and socioeconomic variables. Second, it is widely acknowledged that land development rates are difficult to predict because land development policies are normally inconsistent. Previous studies have predicted future land cover areas according to historical population growth or land development rates, which are impossible to synchronize. This study differed in that we retrieved the quantity of change based on the Markov module. More importantly, this study demonstrated that the Markov-CA model can be applied as a useful tool to support the design of appropriate urban development policies by simulating various scenarios. In addition, the integration of ecosystem services and Markov-CA models was realized to reveal the differences in ecosystem services among the different scenarios, which can support decision making in solving the imbalance between socioeconomic development and ecological protection.

Statistical analysis and spatial mapping have been adopted to reveal the complex interaction relationships. Among them, the correlation coefficient derived from correlation analysis has been commonly adopted to identify the trade-offs and synergies between ecosystem services and quantify their magnitude (Braun et al. 2018; Sylla et al. 2020; Wu et al. 2019). However, the correlation coefficient can only be generated based on the assumption that the relationships between ecosystem services are monotonous, which is false in many cases (Hao et al. 2017). In addition, it is difficult to quantify the complex interaction relationship between ecosystem services through linear thinking because the variations in ecosystem services can be influenced by many factors, including meteorological factors and land cover change. (Bennett et al. 2009). Thus, there is an urgent need to strengthen the quantitative analysis of trade-offs and synergies (Maes et al. 2012). This study demonstrated the effectiveness of the TSD index in quantifying the trade-off and synergy degrees between the various ecosystem services. The TSD index value based on the consideration of the relative change in ecosystem services can be adopted to reveal the direction and magnitude of the interaction relationships between ecosystem services.

### Understanding ecosystem service dynamics and their complex relationships

Our findings suggest that Nanjing city faces major challenges in preventing ecosystem services from degrading within the context of the rapid urbanization process. The loss of ecosystem services due to urban expansion is a common phenomenon around the world. A large number of studies have confirmed that urban expansion can lead to the loss of certain ecosystem services (He et al. 2016; Sun et al. 2018). Because of the rapid urbanization process, cropland, forestland, and grassland were largely occupied by built-up land, which resulted in a decrease in the cropland and vegetated land areas and a loss of ecosystem services. Over the period of 2005–2015, CS, HQ, and air regulation exhibited decrease trends, which are consistent with those of previous studies indicating that urban expansion and ecosystem services are negatively correlated (Braun et al. 2018; Zhang et al. 2019). Furthermore, Seto et al. (2012), Eigenbrod et al. (2011) and Wu et al. (2019) have shown that crop production exhibits a decline trend because of urbanization. In contrast to previous studies, however, crop production increased along with the urbanization process from 2005 to 2015 in Nanjing. Crop production increased along with a decline in the cropland area, which can be explained by the fact that more advanced agricultural technologies and scientific management have been adopted in recent years. As argued by Redhead et al. (2016), SC is highly related to precipitation in the study area, although land cover change affects SC. From 2005 to 2015, WY notably increased because WY is positively correlated with the proportion of built-up land, which has a lower evapotranspiration than vegetated land. Furthermore, our study examined the spatiotemporal dynamics of ecosystem services. The districts of Gaochun, Lishui, and Liuhe, which are far from the city core of Nanjing, attained relatively smaller variations in ecosystem services. The city core districts experienced major changes in ecosystem services.

Although our study proved that continuous urban expansion has imposed major impacts on multiple ecosystem services from 2005 to 2015 in Nanjing, scenario analysis also provides a better understanding of the potential effects of development policies on ecosystem services. Rapid urban expansion and related land cover change in the future will lead to the continuous loss of forestland and cropland, causing negative effects on the supply of ecosystem services (Pham et al. 2019; Zhang et al. 2020). In contrast to past studies that simulated a single scenario in the future, three scenarios were generated to assess the effects of different urban development strategies on ecosystem services. Should the historical land cover trend continue, the BAU scenario simulated the changes in ecosystem services in Nanjing. All of the selected ecosystem services except WY will decrease notably from 2015 to 2030, with a slight increase in WY. Two other scenarios (CP and ER) were simulated under different development policies, such as the CP and ER policies, respectively. Under the CP scenario, the crop production value was notably enhanced due to the strict implementation of CP policies. Compared with the BAU scenario, the trade-offs were reduced. The environmental benefits were emphasized and promoted even more under the ER scenario, which enhanced CS, AP, and HQ. Similar findings were also found in other case studies worldwide, such as the Atlanta Metropolitan area, USA (Sun et al. 2018).

Identifying and analyzing the trade-offs/synergies among multiple ecosystem services under different scenarios can provide support to enhance the decision-making process toward sustainable development (Carreño et al. 2012). The trade-off is an important challenge that decision makers should face to promote the overall ecosystem service value. Deep insights into the mechanisms underlying the trade-offs and synergies among multiple ecosystem services is essential for effective ecosystem service management (Sylla et al. 2020). Both trade-off and synergy relationships were observed. A synergy relationship exists between the regulating and supporting services (HQ). This likely occurs because these ecosystem services strongly rely on a high percentage of vegetated land that can promote these four ecosystem services simultaneously. The synergy among the regulating and supporting services suggests that ER projects can simultaneously improve these services in Nanjing. A trade-off relationship exists between the provisioning services (WY and crop production) and regulating services, which indicates that it is difficult to balance these two types of ecosystem services.

### Implications for land use and ecological management

The accurate recognition of the spatial pattern of ecosystem services and their trade-off and synergy relationships are an important prerequisite for ecological protection and sustainable development, which is conducive to the promotion of human well-being (Longato et al. 2019). In this study, Nanjing city was selected as the study area due to its representation of the typical rapidly developing areas in China, especially in the YRD region, which experienced significant urban expansion at the expense of cropland and vegetated land, as well as degradation of the environment, such as water and heavy haze pollution. To prevent the continuous loss of ecosystem services, the local government proposed several ecological protection plans and policies, including the Ecological Civilization Construction Plan of Nanjing city (2018–2020) and the 13th Five Year Plan for Ecological and Environmental Protection of Nanjing city (2016–2020). Nanjing is identified as a central city in China, an important economic and education base and a comprehensive transportation hub in China. Therefore, Nanjing city faces a great opportunity for development and urbanization, as well as a severe challenge in the sustainable provision of multiple ecosystem services. Analyzing the dynamics of ecosystem services in response to land cover change and simulating the potential effects of different development strategies on ecosystem services can support the decision-making process toward sustained development.

Therefore, two different development scenarios for Nanjing city in 2030 were designed considering ecological management policies, i.e., the CP and ER scenarios. A comparison of the three scenarios indicates that the regulating services can be enhanced at the expense of crop production. Although the two optimized scenarios fail to promote all ecosystem services, they present much smaller losses of ecosystem services than the BAU scenario. The results suggest that ecological land protection and CP should be strictly implemented during the urbanization process to facilitate the functionality of the ecosystem. Comprehensive consideration of various objectives is required to achieve effective urban planning and land use management (Polasky et al. 2011).

The degradation hotspots of ecosystem services were identified in this study and were located in the city core. Although the study demonstrated that rapid urban expansion imposed negative impacts on multiple ecosystem services in Nanjing, it is impossible to limit urban expansion because the city core districts in Nanjing city are socioeconomic centers. To realize the sustainable provision of ecosystem services, it is necessary to balance socioeconomic development and ecological protection. The compact pattern has been recognized as a sustainable development pattern with social and ecological strengths (Mouratidis 2019; Liu et al. 2020). The negative impacts of urban sprawl can be reduced through the intensive use of current urban land resources. Thus, intensive land use management needs to be enforced to prevent urban sprawl and to form compact patterns in the city core.

Human activities and urban planning can potentially impact the formation of trade-off relationships among ecosystem services. For example, Wang et al. (2017) noted that the implementation of the Grain to Green Program has led to the promotion of SC at the expense of crop production. The effective management of multiple ecosystem services and the complex interactions among them during the important urbanization process have become an increasingly important issue for achieving sustainable development (Cord et al. 2017). By weighing the various objectives of ecosystem services, we can reduce the occurrence of conflicts to a certain extent to maximize the overall benefits of ecosystem services and to create a win-win situation of socioeconomic development and ecological protection. Certain environmental protection policies will enhance ecosystem services such as carbon sequestration and oxygen release, hydrological regulation, and soil and water conservation, which may cause a decline in food production. Therefore, from the perspective of food security, the cropland area of Nanjing should be maintained at a certain scale, especially in the subcity districts including the main crop producing areas, which should not be reduced. The red line of cropland should be scientifically identified and located. For cropland that has been reclaimed, measures such as transforming medium- and low-yield fields, using marginal land and improving agricultural facilities should be implemented to ensure the crop supply.

Ecological protection is of equal importance to socioeconomic development. Environmental pollution and ecosystem service degradation can lead to a loss of socioeconomics if ecological protection is ignored. The development and comparison of scenarios indicate that there is an urgent need to protect ecological land to mitigate the negative effects of urban expansion on ecosystem services. The policy of the red line of ecological land should be strictly implemented to protect the important ecological land area. The area of ecological land is decreasing, and the quality of ecosystem service functions is becoming highly variable due to rapid urbanization. Ecological corridors play a key role in enhancing the regulation of ecosystem services, including AP, SC, climate regulation, and biodiversity. Therefore, ecological corridors should be established and protected by implementing protection policies. Because of the relatively scarce land sources in the city core districts, ecological corridors should be established along primary roads and rivers to promote ecosystem services. The city core areas of Nanjing city attained very low values of the regulating and supporting ecosystem services, despite the high population density and economic level, and therefore strongly relied on the suburban areas of Nanjing where high regulating, supporting, and provisioning service values were observed. People who live in the city core therefore required the ecosystem services generated in suburban areas. Decision makers should implement certain effective measures to protect the important ecological patches from being rapidly urbanized because they are an important source of ecosystem services.

**Fig. 7:**
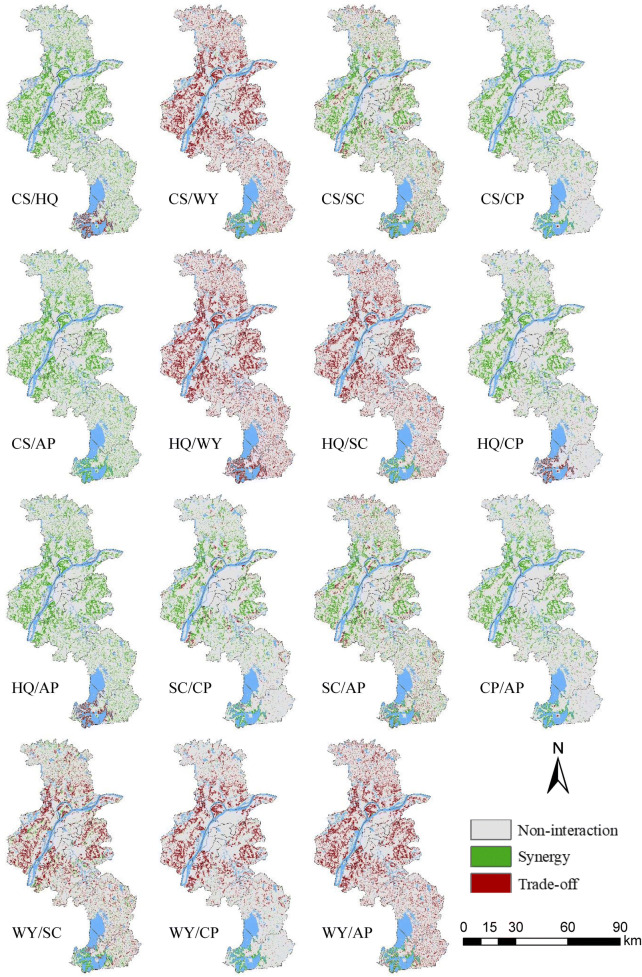
Spatial patterns of the trade-offs/synergies between ecosystem services under the BAU scenario

**Fig. 8:**
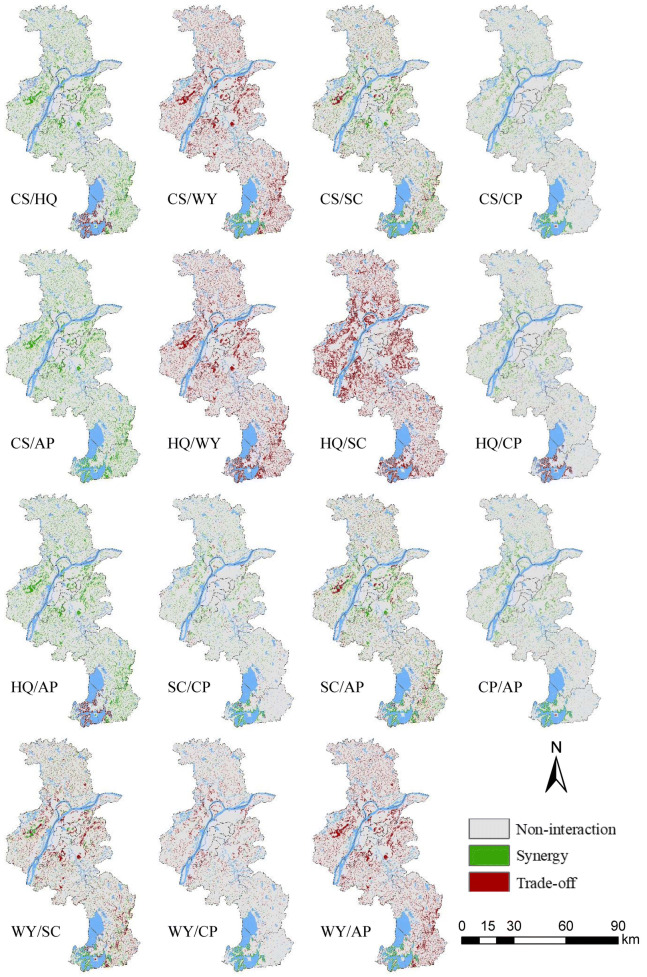
Spatial patterns of the trade-offs/synergies between ecosystem services under the CP scenario

**Fig. 9:**
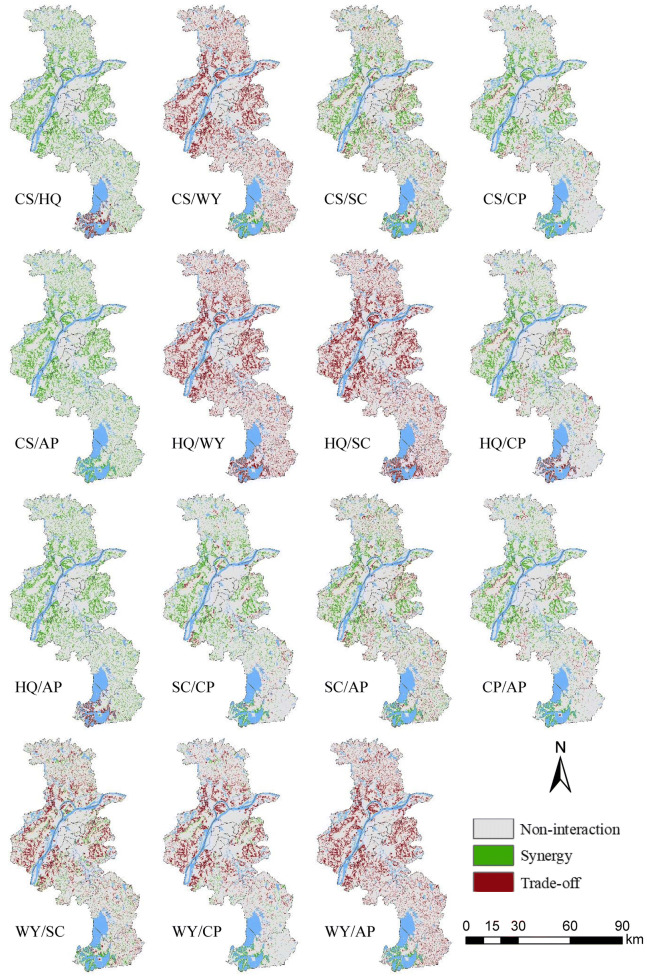
Spatial patterns of the trade-offs/synergies between ecosystem services under the ER scenario

It is worth noting that ecosystem services are not distributed evenly on a spatial basis (Zhang et al. 2020) and spatial heterogeneity is inherent within the synergy and trade-off. As illustrated in Figs. 7, 8, and 9, our findings clearly indicate the areas where the synergy and tradeoffs occurred. Spatial patterns of trade-offs and synergies were highly heterogeneous and diverse. For effective ecological management and planning, the decision makers need to consider not only the overall relationship between ecosystem services, but also their spatial patterns. Although the global relationship between CS and HQ was synergy, both trade-off and synergy between CS and HQ under the ER scenario can be observed in Fig. 9. Therefore, it is of great importance to formulate and implement ecological protection and planning polices by involving the spatial differences of trade-offs/synergies between ecosystem services.

### Limitations

In this study, there are limitations. Due to data availability, land cover data with a relatively low spatial resolution were analyzed in this study, which limits the ability to fully understand land cover change. Although many types of ecosystem services are important for human well-being and sustainable development, only five ecosystem service types were selected and assessed because of the lack of suitable assessment methods and data. Furthermore, land use change is a complicated process that involves various driving factors, e.g., policies, development strategies, and uncertainties. However, these factors were not considered in the simulation models in this study. Nevertheless, we analyzed the path of optimizing the land spatial patterns to maintain key ecosystem services in Nanjing in this study. The results can provide a crucial basis for urban planning and ecosystem management to achieve regional sustainable development. The ES models adopted in this study for evaluating ecosystem service values needs to be further improved in future study. The carbon storage module in InVEST simplifies the carbon cycle and assumes that no land cover types are gaining or losing carbon over time. However, some areas are undergoing natural succession. This can be improved by conducting field survey and dividing land cover types into different age classes. For estimating soil conservation, the module is very sensitive to the parameters C and P, which are derived from relevant studies. Local empirical data could be used for improving the accuracy of evaluation. Air purification was assessed based on a simplicisitic approach. However, PM10 removal is strongly depended on multiple factors, including leaf area, tree species, background air pollutant concentrations which were not involved in this study. More comprehensive approaches and realistic parameters for estimating ecosystem services need to be established in future studies.

## Conclusions

Ecosystem services are of great importance to achieve sustainable development and promote human well-being. In this study, we examined the spatiotemporal dynamics of six types of ecosystem services in Nanjing city over the period of 2005–2015. From 2005 to 2015, the ecosystem services of CS, HQ, and AP decreased, while those of crop production, WY, and SC increased. Three different scenarios were simulated to assess the impacts of alternative development strategies on the ecosystem services from 2015 to 2030. The ecosystem services values under the three scenarios all exhibit different trends and trade-off/synergy relationships. The crop production service can be effectively protected under the CP scenario, as crop production will increase from 2015 to 2030. Moreover, the ER scenario performs better than the other two scenarios in protecting the regulating and supporting services. However, it improves most ecosystem services at the expense of crop production. Under the BAU scenario, the ecosystem services will notably decline except for WY, which likely occurs due to urban expansion encroaching on cropland and forestland. In the future, the loss of ecosystem services caused by urban expansion might continue if there are no major policy changes. To promote all ecosystem services and minimize trade-offs, certain development strategies, including the construction of ecological corridors and compact development, are suggested to be implemented to increase the regulating services without threatening the other ecosystem services or occupying additional land resources.

## Electronic supplementary material

ESM 1 (DOCX 18 kb)
