# Supplementary material for: Investigating spatiotemporal dynamics and trade-off/synergy of multiple ecosystem services in response to land cover change: a case study of Nanjing city, China
Source: Environ Monit Assess. 2020 Oct 13;192(11):701. doi: 10.1007/s10661-020-08663-x (PMC7554017; doi:10.1007/s10661-020-08663-x)
Supplement: Supplementary file 1 — (DOCX 18 kb) [file 10661_2020_8663_MOESM1_ESM.docx]

Table A.1 Biophysical table in water yield module for Nanjing, China.

| LULC type | Kc | root_depth | usle_c | usle_p | sedret_eff | load_n | eff_n | load_p | eff_p | LULC_veg | crit_  len_p | crit_  len_n | load_  subsurface_n | load_  subsurface_p | proportion_  subsurface_n |
| --- | --- | --- | --- | --- | --- | --- | --- | --- | --- | --- | --- | --- | --- | --- | --- |
| Crop land | 0.65 | 2000 | 0.25 | 0.4 | 0.25 | 5.3 | 0.25 | 1.5 | 0.25 | 1 | 150 | 150 | 0.53 | 0.15 | 0.3 |
| Forest | 1 | 7000 | 0.003 | 0.2 | 0.6 | 1.8 | 0.8 | 0.011 | 0.8 | 1 | 150 | 150 | 0.18 | 0.0011 | 0 |
| Grassland | 0.65 | 1700 | 0.008 | 0.2 | 0.4 | 11 | 0.4 | 1.5 | 0.4 | 1 | 150 | 150 | 1.1 | 0.15 | 0 |
| Waterbody | 1 | 500 | 0.001 | 0.001 | 0.05 | 0.001 | 0.05 | 0.001 | 0.05 | 0 | 150 | 150 | 0.0001 | 0.0001 | 0 |
| Built-up land | 0.3 | 500 | 0.001 | 0.001 | 0.05 | 9 | 0.05 | 2.5 | 0.05 | 0 | 150 | 150 | 0.9 | 0.25 | 0 |
| Bare land | 0.2 | 10 | 0.25 | 0.01 | 0.2 | 4 | 0.05 | 0.001 | 0.05 | 0 | 150 | 150 | 0.4 | 0.0001 | 0 |

Table A.2 Carbon density per unit area of different land cover types in Nanjing, China (Unit: Mg/ha)

| Land cover type | Aboveground | Belowground | Soil organic | Dead organic |
| --- | --- | --- | --- | --- |
| Cropland | 22.4 | 80.7 | 108.4 | 5 |
| Forest | 44.8 | 128.6 | 140.5 | 46.1 |
| Grassland | 35.3 | 86.5 | 99.9 | 2 |
| Waterbody | 0 | 0 | 119 | 0 |
| Built-up land | 1.9 | 50.2 | 94.2 | 0 |
| Bareland | 20.6 | 70.3 | 97.7 | 0 |

Table A.3 Biophysical table in soil conservation module in Nanjing, China

| Land cover type | lucode | usle_c | usle_p |
| --- | --- | --- | --- |
| Crop land | 1 | 0.031 | 0.4 |
| Forest | 2 | 0.006 | 1 |
| Grassland | 3 | 0.001 | 0.8 |
| Waterbody | 4 | 0.001 | 0.001 |
| Built-up land | 5 | 0.001 | 0.001 |
| Bare land | 6 | 0.01 | 0.9 |

Table A.4 Parameters for evaluating air purification in Nanjing, China (Unit: kg/ha)

| Items | Crop land | Forest | Grassland | Waterbody | Built-up land | Unused land |
| --- | --- | --- | --- | --- | --- | --- |
| PM10 capture | 9.2 | 62 | 27 | 0 | 0 | 0 |

Table A.5 Parameters for habitat quality in Nanjing, China

|  |  | **Threats** | | | | | |
| --- | --- | --- | --- | --- | --- | --- | --- |
|  |  | Habitat | Crop | Built-up | National way | Highway | Provincial way |
| **The properties of threats** | Max_Distance | - | 8 | 10 | 3 | 1 | 1 |
|  | Weight | - | 0.7 | 1 | 1 | 0.7 | 0.7 |
|  | Decay | - | linear | exponential | linear | linear | linear |
| **Sensitivity of different land cover types** | Cropland | 0.3 | 0.35 | 0.3 | 0.2 | 0.2 | 0.1 |
|  | Forest | 0.5 | 0.55 | 0.5 | 0.4 | 0.4 | 0.3 |
|  | Grassland | 0.3 | 0.35 | 0.3 | 0.2 | 0.2 | 0.1 |
|  | Waterbody | 1 | 0.7 | 0.9 | 0.7 | 0.6 | 0.6 |
|  | Built-up | 0 | 0 | 0 | 0 | 0 | 0 |
|  | Bareland | 0 | 0 | 0 | 0 | 0 | 0 |
